# Artificial Intelligence-Assisted Versus Traditional Learning and Long-Term Knowledge Retention Among Undergraduate Medical Students: A Sequential, Explanatory Mixed-Methods Study

**DOI:** 10.7759/cureus.114362

**Published:** 2026-08-11

**Authors:** Thenrajan Padmavathi, Paulraj Rajavel Murugan, K R Sethuraman, Judson Neslin J

**Affiliations:** 1 Pharmacology, Kanyakumari Government Medical College, Kanyakumari, IND; 2 Health Professions Education, Sri Balaji Vidyapeeth University, Puducherry, IND; 3 General Medicine, Government Thoothukudi Medical College, Thoothukudi, IND; 4 Community Medicine, Kanyakumari Government Medical College, Kanyakumari, IND

**Keywords:** artificial intelligence, bloom's 2 sigma problem, generative ai, long-term memory retention, medical education

## Abstract

Background

Artificial intelligence (AI) is increasingly being integrated into medical education to support self-directed learning through personalized explanations and immediate feedback. Although AI has been shown to improve learning outcomes, its effect on long-term memory retention remains unclear. This study compared long-term memory retention following AI-assisted learning and traditional learning among undergraduate medical students and explored students' perceptions regarding AI-assisted learning.

Methods

A prospective, within-subject, sequential, explanatory mixed-methods study was conducted among 149 second-year MBBS students at Kanyakumari Government Medical College, Tamil Nadu, India. Students completed two learning sessions: traditional learning (aminoglycosides) and AI-assisted learning (quinolones), each consisting of a pre-test, a 15-minute introductory lecture, one hour of self-directed learning, and an immediate post-test. Knowledge retention was assessed using parallel Very Short Answer Question (VSAQ) tests at one week and one month. Quantitative data were analyzed using paired t-tests and repeated-measures analysis of variance (ANOVA). Subsequently, focus group discussions were conducted among students with high and low retention scores, and qualitative data were analysed using thematic analysis.

Results

Both learning methods significantly improved immediate post-test scores. However, AI-assisted learning was associated with significantly higher knowledge retention at 1 week (75.5% vs. 57.9%) and 1 month (61.4% vs. 35.9%) compared with traditional learning (p<0.001). Repeated-measures ANOVA showed significant effects of learning method (F=181.66, p<0.001), assessment time (F=625.73, p<0.001), and their interaction (F=62.23, p<0.001). A significantly greater proportion of students exceeded a Bloom-inspired heuristic benchmark for high memory retention following AI-assisted learning (43.4% vs. 3.0%). This benchmark was conceptual and exploratory rather than an exact replication of Bloom's original Two Sigma methodology. Of 149 enrolled students, 115 attended the pre-test in both modalities and formed the base analytic cohort; 95 students (82.6% of this cohort) had complete data across all 4 assessment waves, were included in the repeated-measures analysis, and did not differ from non-completers on baseline scores. Four themes emerged from the qualitative analysis: AI as a personalized tutor, enhanced conceptual understanding, visual learning and memory retention, and cognitive offloading.

Conclusion

AI-assisted learning was associated with higher long-term memory retention compared with traditional learning under the specific, non-randomized conditions investigated; this association does not establish that AI-assisted learning caused the improvement. Students' perceptions of personalized, interactive explanations and active engagement appeared linked to improved retention, whereas passive reliance on AI-generated responses was associated with cognitive offloading. Confirmation of these associations, and of any causal role for AI-assisted learning, will require randomized crossover trials using equivalent topics, standardized AI platforms, and validated assessments.

## Introduction

Artificial intelligence has rapidly transformed educational practices, creating new opportunities for teaching and learning. It provides immediate access to information, personalized explanations, and adaptive learning experiences. Medical education is not exempt from this transformation and has increasingly adopted generative artificial intelligence (GenAI) to support teaching, learning, and self-directed education [1]. Various GenAI platforms, such as ChatGPT, Gemini, and NotebookLM, are increasingly being used by medical students to supplement their traditional learning resources [2].

GenAI can support learning in different ways. AI-assisted learning refers to the use of GenAI tools, such as ChatGPT and Gemini, to facilitate learning through explanations, summaries, quizzes, and clarification of concepts [3]. In contrast, AI tutoring involves AI functioning as an individualized instructional tutor by providing adaptive guidance and personalized feedback [4]. Unrestricted use of generative AI, however, refers to open-ended querying of AI systems, where learners primarily seek answers or generate content [5]. The present study focuses on AI-assisted learning using generative AI platforms rather than dedicated AI tutoring systems. This distinction is important because these approaches differ in their level of instructional support, learner engagement, and potential influence on learning and long-term memory retention.

According to Bloom's landmark work on the "2 Sigma Problem," students receiving one-to-one tutoring performed approximately two standard deviations (2 SD) better than those receiving conventional instruction [6]. However, the widespread implementation of one-to-one tutoring is challenging because of its substantial resource requirements, including faculty time, infrastructure, and financial costs. Consequently, AI-based educational systems, including AI tutoring, have been proposed as a scalable alternative capable of providing individualized instruction, immediate feedback, and adaptive explanations. Memory retention is a critical component of successful medical education. Newly acquired information is easily forgotten if it is not reinforced through retrieval practice and elaborative learning according to the Ebbinghaus forgetting curve [7]. Educational interventions are therefore essential for enhancing memory consolidation. However, during AI-assisted learning, information is readily available, and there is a potential risk of cognitive offloading [8,9]. Learners may rely on AI-generated responses rather than actively processing information. Such dependence may reduce retrieval effort and impair long-term memory consolidation.

Despite the increasing adoption of GenAI in medical education, evidence regarding its influence on long-term memory retention remains limited and inconclusive. While AI-assisted learning offers personalized explanations and rapid access to information, concerns have been raised regarding cognitive offloading and reduced retrieval effort, which may influence memory consolidation.

Based on the existing evidence, we hypothesized that undergraduate medical students using AI-assisted learning would demonstrate significantly different long-term memory retention compared with those using traditional learning methods. We further anticipated that students' perceptions would provide contextual insights into the perceived benefits, limitations, learning behaviours, and cognitive engagement associated with AI-assisted learning, thereby helping to explain the quantitative findings. So, a sequential mixed-method study is needed, which could capture phenomenological qualitative data to explain the quantitative findings.

Accordingly, the present study aimed to compare immediate knowledge acquisition and long-term memory retention (at one week and one month) following AI-assisted learning and traditional learning among undergraduate medical students, and to explore students' perceptions regarding AI-assisted learning to contextualize these quantitative findings. One-month retention was pre-specified as the primary outcome, consistent with the study's focus on long-term retention, with immediate post-test and one-week retention scores designated as secondary outcomes.

## Materials and methods

This prospective, within-subject, sequential, explanatory mixed-methods study was conducted among second-year MBBS students in the Department of Pharmacology at Government Kanyakumari Medical College, Tamil Nadu, India. The study compared traditional learning with artificial intelligence-assisted learning. Immediate knowledge acquisition and long-term knowledge retention were compared between these two learning methods, followed by students' perceptions of AI-assisted learning through focus group discussions. Ethical approval was obtained from the Institutional Ethics Committee, Kanyakumari Government Medical College (F-002/2026, dt 07/03/2026) before commencement of the study, and written informed consent was obtained from all participants.

All eligible second-year MBBS students who provided informed consent were included in the study, and those absent during any scheduled learning session or assessment were excluded during analysis. A total of 149 students participated in the quantitative phase. No prior sample size was calculated, as the study included the entire eligible cohort.

Quantitative methodology

The study was carried out in two instructional sessions. During the first session, students studied aminoglycoside pharmacology using conventional self-directed learning supported by standard pharmacology textbooks following a 15-minute introductory lecture. During the second session, students studied quinolone pharmacology using GenAI intelligence platforms, including ChatGPT, Gemini, or NotebookLM, according to their individual preference. Students were instructed not to use GenAI tools during the traditional-learning session, so that the traditional condition remained free of AI assistance; adherence to this instruction was not independently monitored. By protocol design, the second instructional session and its associated assessment schedule (immediate, one-week, and one-month) commenced only after the first session's complete assessment schedule had concluded, resulting in a minimum inter-session interval of approximately one month. Each assessment (pre-test, immediate post-test, 1-week retention test, and 1-month retention test) consisted of 15 Very Short Answer Questions (VSAQs). Students were allotted 30 minutes to complete each assessment under supervised examination conditions. The time allocation provided approximately two minutes per question, which was considered adequate for reading, recalling, and recording brief responses. All participants completed the assessments within the allotted time, and no additional time was required. The same time limit was maintained across all assessment sessions to ensure uniform testing conditions. To ensure comparability between instructional approaches, identical learning objectives, study duration, assessment format, and structured study guides were used for both sessions. The study guide given included: 1. Classify drugs, 2. Explain mechanisms of action and pharmacological effects, 3. Identify therapeutic indications with appropriate rationale, and 4. Recognize important adverse drug reactions, ensuring that only the learning modality differed between the two instructional approaches. The study guide was used solely to standardize the learning outcomes across both instructional approaches and did not contain summary notes, key facts, mnemonics, or other memory aids. No supplementary revision handouts or reinforcement materials were provided to either group.

In addition to the study guide, students were sensitised to effective prompting strategies during their self-learning. They were instructed how to frame specific questions, request in-depth explanations, and use prompts for further follow-up. This was done to ensure that the differences in learning outcomes would be attributable to the AI tools rather than the prompting skill of the student.

The VSAQ instrument was developed by the investigators based on the predefined learning objectives of the topics quinolones and aminoglycosides. The initial pool of questions was reviewed by a panel of experts comprising three senior pharmacology faculty and one medical education unit faculty. The instrument was validated for content, relevance, and clarity. Based on their opinion, ambiguous items were removed, and the final instrument was constructed with 15 VSAQs for each topic. The instrument demonstrated a good internal consistency (Cronbach's alpha: 0.79).

Two topic-specific VSAQ assessments were developed for quinolones and aminoglycosides. To ensure comparability, both assessments were constructed using the same test blueprint, with identical numbers of items (15 VSAQs), comparable cognitive levels based on Bloom's taxonomy, similar content coverage, and equivalent marks. The questions were reviewed by subject experts to ensure comparable difficulty and alignment with the intended learning outcomes.

Each VSAQ carried two marks. Each VSAQ was scored by a three-point rubric (0-not correct or blank, 1-partial, 2-correct). No negative marking was applied. The maximum score for each assessment was 30. The same rubric was used in all the assessments. The answer key included predetermined acceptable answers.

To minimize assessment bias, all answer sheets were anonymized and coded before evaluation. Assessors were blinded to the students' identities, study group, and assessment time point (pre-test, post-test, one-week retention, or one-month retention) during scoring.

Scoring was carried out by trained pharmacology faculty against the predefined rubric and answer key described above, which was designed to standardize scoring decisions and minimize subjective judgment. Formal statistical assessment of inter-rater reliability (e.g., Intraclass Correlation Coefficient) was not performed in this study; this is acknowledged as a limitation, and future iterations of this assessment protocol should incorporate a dual-rating design with a calculated reliability statistic to further strengthen scoring rigor.

Because not all students had a score in both learning modalities at every assessment time point, paired t-tests at each time point were restricted to students with matched, non-missing scores in both modalities at that time point; a student missing a score in either modality at a given time point was excluded from that specific paired comparison only, and sample sizes therefore varied across time points. Repeated-measures analysis of variance (ANOVA) was further restricted to the subset of students with complete, matched observations across all four time points in both modalities. Quantitative data were analyzed using IBM SPSS Statistics for Windows, Version 25.0 (IBM Corp., Armonk, NY, USA), which included descriptive statistics, paired t-tests, Cohen's d effect sizes, and repeated-measures ANOVA. Mauchly's test was used to assess the sphericity assumption for repeated-measures ANOVA; where the assumption was violated, Greenhouse-Geisser corrected degrees of freedom were applied. Bonferroni correction was applied across the four paired timepoint comparisons to account for multiple testing. Cohen's d values were interpreted as small (0.2), moderate (0.5), and large (0.8). Knowledge retention was expressed as the percentage of immediate post-test scores retained at one week and one month, while retention decay was calculated as: Retention Decay (%) = ((Immediate Post-test Score − Follow-up Score) / Immediate Post-test Score) × 100, with lower values indicating slower forgetting. Repeated-measures ANOVA included only participants with complete observations across all assessment time points, and statistical significance was set at p < 0.05.

Qualitative methodology

Following completion of the quantitative phase, a qualitative exploration was undertaken, which adhered to the Consolidated Criteria for Reporting Qualitative Research (COREQ) guidelines [10]. Focus group discussions (FGDs) were conducted to assess the in-depth understanding of students' experiences with AI-assisted self-directed learning and to complement the quantitative findings. Each of the three FGDs conducted comprised six students and was conducted by a trained faculty member from the Department of Community Medicine who had sufficient expertise in qualitative research, who was not teaching this cohort of students, and who did not have any pre-fixed assumptions about AI-assisted learning. This selection of faculty was meant to reduce the power discrepancies between students and the data collector. Students were selected through purposive sampling with varying levels of knowledge retention (high and low retention). A semi-structured interview guide, developed based on the study objectives and relevant literature, was used to facilitate the discussions (Appendices). The FGDs were conducted in the pharmacology department seminar hall, and each session lasted about 40 minutes. All FGDs were audio-recorded by a non-teaching staff member, with participants' consent, transcribed verbatim, anonymised, and checked for transcription accuracy before analysis.

The transcripts were analysed using NVivo 14 (Lumivero, Denver, CO, USA). Data were read repeatedly for familiarization, then the data coding process was started, followed by categorization based on concept similarity. Related categories were synthesized into broader themes, which represented students' views on AI-assisted learning. Two investigators independently reviewed and coded the transcripts. The discrepancies during the coding were resolved through discussion until consensus was achieved. Data collection continued until thematic saturation was reached, with no new codes emerging. Investigator triangulation and researcher reflexivity were maintained throughout the analytical process to minimize the potential researcher bias and increase the trustworthiness of the analysis process.

## Results

Participant flow and sample characteristics

**Figure 1:**
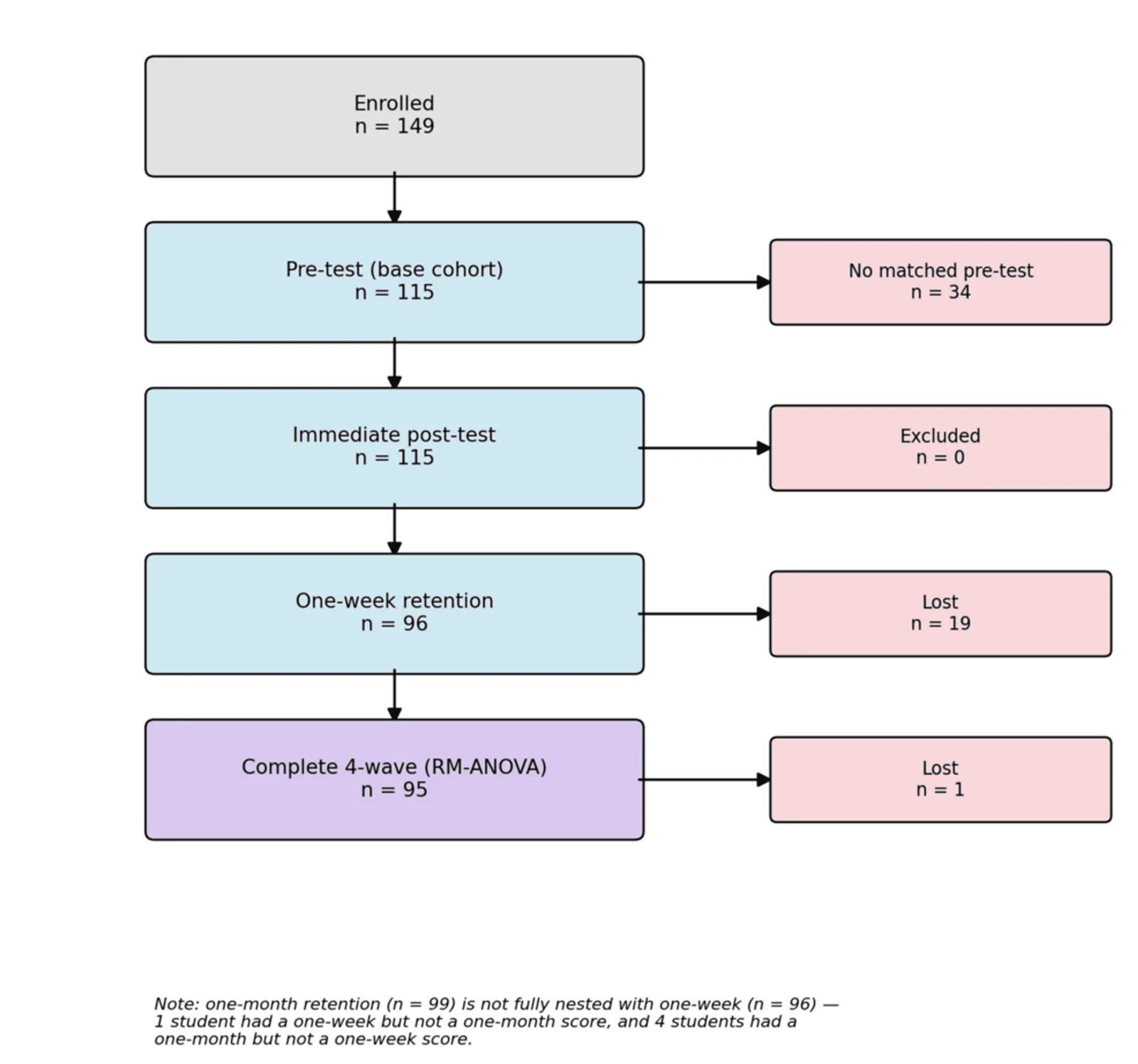
Participant flow diagram showing the pre-test attendance base cohort and sequential attrition to the complete-case sample used in repeated-measures analysis

Of 149 students enrolled in the study, 115 (77.2%) attended pre-test assessment in both the traditional and AI-assisted learning modalities and formed the base analytic cohort for all paired comparisons; the remaining 34 students lacked a matched pre-test score in one or both modalities and were excluded from further paired analysis. Within this base cohort, all 115 students (100%) had immediate post-test scores available in both modalities. Attrition occurred progressively thereafter: 96 students (83.5%) completed one-week retention testing in both modalities, and 99 students (86.1%) completed one-month retention testing in both modalities. Ninety-five students (82.6% of the base cohort) had complete, matched data across all four assessment waves in both learning modalities and were included in the repeated-measures analysis (Figure 1).

Missing data assessment

To evaluate whether attrition after pre-test was systematically related to baseline performance, students with complete four-wave data (n = 95) were compared with those who attended pre-test but did not complete all subsequent assessments (n = 20). Baseline pre-test scores did not differ significantly between completers and non-completers in either the traditional (6.37 ± 3.72 vs. 6.20 ± 4.67; t = 0.15, p = 0.881) or AI-assisted (4.73 ± 2.49 vs. 4.75 ± 3.09; t = −0.03, p = 0.975) modality (Table 1). This finding suggests that attrition was unrelated to baseline knowledge and reduces the likelihood that missing data at later timepoints biased the observed retention effects.

**Table 1:** Baseline comparison of completers versus non-completers

Group	Traditional pre-test (Mean ± SD)	AI-assisted pre-test (Mean ± SD)
Completed all 4 waves (n = 95)	6.37 ± 3.72	4.73 ± 2.49
Incomplete after pre-test (n = 20)	6.20 ± 4.67	4.75 ± 3.09
Welch's t	0.15	−0.03
p value	0.881	0.975

Comparison of traditional and AI-assisted learning across assessment timepoints

Paired comparisons within the base cohort showed that AI-assisted learning produced significantly lower pre-test scores than traditional learning (mean difference = −1.61, p < 0.001), but significantly higher scores at all three subsequent timepoints, with the magnitude of advantage increasing through one-week retention before narrowing slightly by one month (Table 2).

**Table 2:** Paired comparison of traditional versus AI-assisted learning outcomes Note: One-week (n = 96) and one-month (n = 99) samples are not fully nested — one student had a one-week but not a one-month score, and four students had a one-month but not a one-week score, reflecting minor session-specific attendance variation.

Assessment	Traditional (Mean ± SD)	AI-assisted (Mean ± SD)	Mean diff. (AI − Trad)	95% CI	t (df)	p	Cohen's d	n
Pre-test	6.34 ± 3.88	4.73 ± 2.59	−1.61	−2.18 to −1.04	−5.62 (114)	<0.001	0.52	115
Immediate post-test	21.68 ± 3.80	25.11 ± 4.32	3.43	2.50 to 4.37	7.29 (114)	<0.001	0.68	115
One-week retention	12.54 ± 3.52	19.23 ± 5.38	6.69	5.71 to 7.66	13.60 (95)	<0.001	1.39	96
One-month retention	7.78 ± 4.02	15.49 ± 8.15	7.71	6.19 to 9.23	10.06 (98)	<0.001	1.01	99

Repeated-measures analysis across time and modality

A two-way repeated-measures ANOVA (Method × Time) was conducted on the 95 students with complete data across all four waves in both modalities (Table 3). Mauchly's test indicated that the assumption of sphericity was violated for both the main effect of time (χ²(5) = 236.18, p < 0.001) and the Method × Time interaction (χ²(5) = 222.28, p < 0.001); Greenhouse-Geisser corrected degrees of freedom are therefore reported. Significant main effects were observed for learning method (F(1, 94) = 181.66, p < 0.001, partial η² = 0.659) and time (F(1.54, 144.88) = 625.73, p < 0.001, partial η² = 0.869). Critically, a significant Method × Time interaction was observed (F(1.46, 137.43) = 62.23, p < 0.001, partial η² = 0.398), indicating that the magnitude of the AI-assisted learning advantage varied significantly across the retention interval, rising sharply between immediate post-test and one-week retention before narrowing at one-month (Table 4).

**Table 3:** Repeated-measures ANOVA results (n = 95) ANOVA: analysis of variance

Effect	Uncorrected F (df)	Greenhouse-Geisser (GG) ε	GG-corrected df	GG-corrected p	Partial η²
Method	181.66 (1, 94)	—	—	<0.001	0.659
Time	625.73 (3, 282)	0.514	(1.54, 144.88)	<0.001	0.869
Method × Time	62.23 (3, 282)	0.487	(1.46, 137.43)	<0.001	0.398

**Table 4:** Cell means for Method × Time interaction (n = 95, complete cases)

Modality	Pre-test	Immediate post-test	One-week retention	One-month retention
Traditional	6.37	21.75	12.56	7.86
AI-assisted	4.73	25.45	19.21	15.66

Retention percentage and decay analysis

Knowledge retention gradually declined over time following both learning methods, but the decline was consistently smaller following AI-assisted learning. Within the base cohort, students retained 75.5% of immediate post-test knowledge at one week following AI-assisted learning compared with 57.9% following traditional learning. At one month, AI-assisted learning retained 61.4% of acquired knowledge, whereas traditional learning retained only 35.9%, corresponding to a one-month retention decay of 38.6% for AI-assisted learning versus 64.1% for traditional learning (Table 5).

**Table 5:** Retention and decay analysis (base cohort) Note: Retention and decay percentages were calculated at the participant level, using the mean immediate post-test score of the same matched subset of students available at each follow-up timepoint (n = 96 at one week; n = 99 at one month) as the denominator, rather than the overall base-cohort mean (n = 115).

Outcome	n	Traditional (base cohort)	AI-assisted (base cohort)
Immediate post-test (mean), one-week subset	96	21.68	25.47
One-week score (mean)	96	12.54	19.23
One-week knowledge retained (%)	96	57.9	75.5
Immediate post-test (mean), one-month subset	99	21.70	25.25
One-month score (mean)	99	7.78	15.49
One-month knowledge retained (%)	99	35.9	61.4
One-month retention decay (%)	99	64.1	38.6

Bloom-inspired heuristic benchmark analysis

One-month retention scores following traditional learning, restricted to the base cohort with matched observations in both modalities (n = 99), were used to derive a Bloom-inspired heuristic benchmark as the traditional group's mean plus two standard deviations (7.78 + 2 × 4.02 = 15.82 marks). This benchmark was used solely as a descriptive, exploratory indicator of exceptionally high knowledge retention within the present cohort and should not be interpreted as a direct replication of Bloom's original 2 Sigma methodology, which compared individualized human tutoring against traditional classroom instruction rather than a within-cohort statistical threshold. Using this benchmark, 3.0% (3/99) of students following traditional learning and 43.4% (43/99) following AI-assisted learning exceeded the threshold, indicating a substantially greater proportion of students reaching exceptionally high one-month retention following AI-assisted learning (Table 6).

**Table 6:** Bloom-inspired heuristic benchmark analysis (base cohort)

Parameter	Value
Mean one-month retention score, Traditional (base cohort)	7.78
Standard deviation (SD)	4.02
Bloom-inspired benchmark (Mean + 2 SD)	15.82 marks
Students assessed at one month (base cohort), n	99
Students achieving ≥ 15.82 marks, Traditional	3 (3.0%)
Students achieving ≥ 15.82 marks, AI-assisted	43 (43.4%)

Qualitative findings

**Figure 2:**
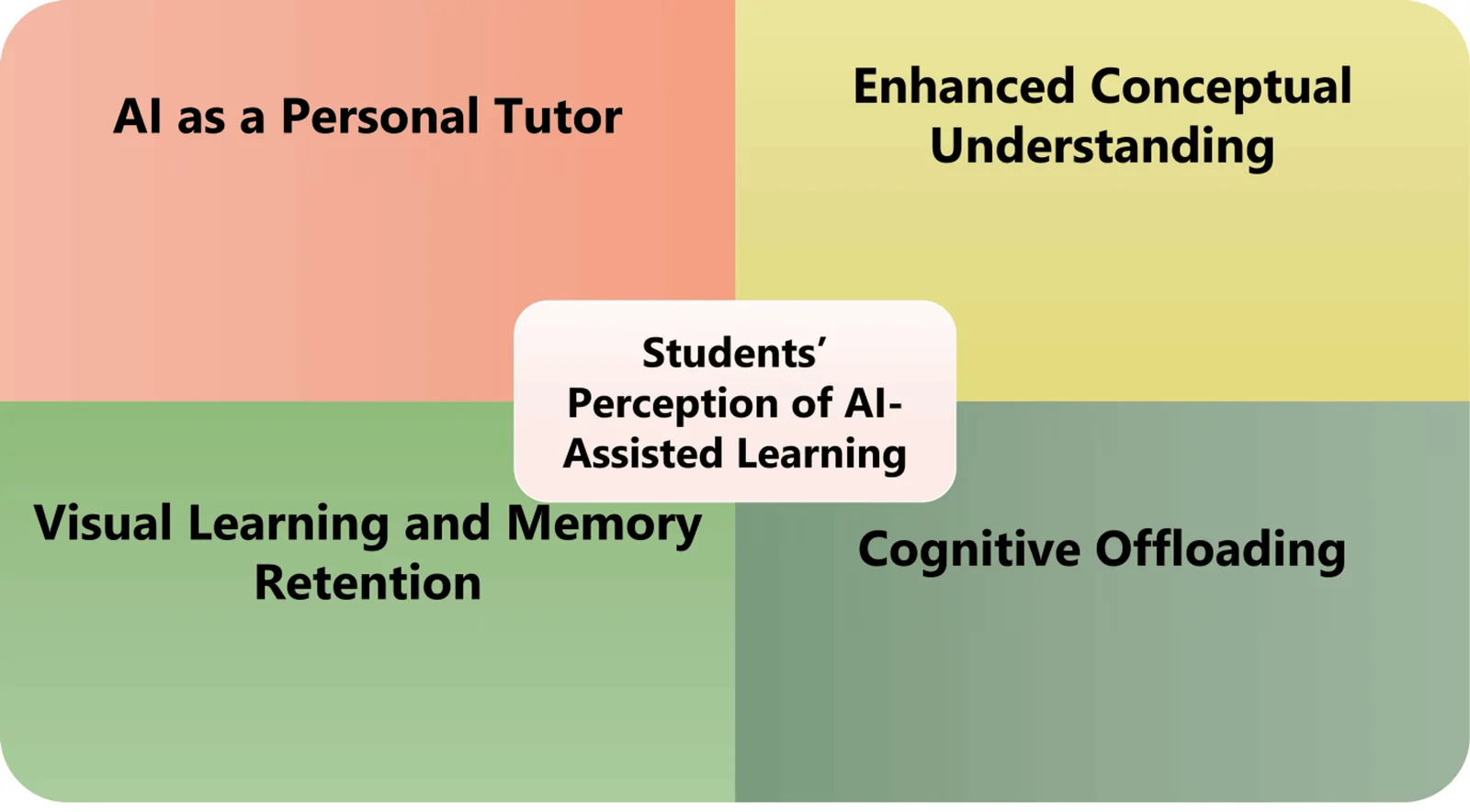
Thematic Analysis of Student Perception

Focus group discussions were conducted among students with high and low long-term retention scores to explore their experiences with AI-assisted learning. Thematic analysis identified four major themes: AI as a personalized tutor, enhanced conceptual understanding, visual learning and memory retention, and cognitive offloading (Figure 2).

Theme 1: AI as a Personalized Tutor

Some of the students’ quotes were:

“It was like a personal teacher who cleared my doubts; I didn’t feel shy to ask any silly question.”

“I was able to ask any number of clarifications, which made me remember more than using a textbook, which is static.”

Participants consistently mentioned AI as functioning similarly to a personal tutor. It provided immediate feedback, individualized explanations, and clarification. Students welcomed the ability to ask repeated questions and receive explanations adapted to their learning needs.

Theme 2: Enhanced Conceptual Understanding

Repeated explanations, flowcharts, concept maps, comparisons, and clinical correlations by the AI models were very useful to understand the concepts of the learning process. These features helped them integrate basic pharmacological concepts with their clinical applications. Some students’ quotes were:

“Theory and clinical applications connect was better with AI.”

“Flow charts made me connect the concepts easily.”

Theme 3: Visual Learning and Memory Retention

Participants repeatedly emphasized the role of AI-generated visual representations in improving memory retention. Tables, pictures, flowcharts, and mnemonics were commonly used as retrieval cues during the delayed assessments. Some students’ quotes were:

“Mechanism of action and adverse effects with pictures by visual memory.”

“Tables and pictures helped me remember the topic better.”

Theme 4: Cognitive Offloading

A few students relied only on AI-generated information; they didn’t actively process it. For such students, there was a challenge of cognitive offloading. They mentioned:

“I only read the answers.”

“I copied answers from AI.”

“Since answers were fast, I just had a quick glance.”

In contrast, students with better long-term retention described dynamically interacting with AI by asking many repeated and follow-up questions. They have done an active process of learning with AI, for example:

“Repeated prompts at my own pace made me remember because it became active learning.”

Table 7 briefly describes the four themes. 

**Table 7:** Themes, codes, and representative quotations from focus group discussions

Theme	Codes	Representative quotation
AI as a personalized tutor	Immediate feedback; Individualized explanations; Clarification of doubts; Self-paced learning	"It was like a personal teacher who cleared my doubts; I didn't feel shy to ask any silly question."
Enhanced conceptual understanding	Flowcharts; Concept maps; Clinical correlations; Simplified explanations; Comparative learning	"Flow charts made me connect the concepts easily."
Visual learning	Visual memory; Tables; Diagrams; Mnemonics; Retrieval cues	"Tables and pictures helped me remember the topic better."
Cognitive offloading	Passive learning; Copy-paste behaviour; Reduced critical thinking; Surface learning	“I only read the answers.” “I copied answers from AI.”

## Discussion

In the present study, students achieved significantly higher immediate post-test scores in both traditional and AI-assisted learning. When assessing retention scores at about one week and one month, a significant difference was observed between the two learning methods. AI-assisted learning was found to have higher retention scores. Kalam et al. have also reported high immediate academic performance among students using ChatGPT, but their results are contradictory to the present study for memory retention [11]. Our findings more closely align with the results of Daniel et al., who demonstrated higher memory retention in the students' ChatGPT-integrated knowledge retrieval tutorials [12]. So, the higher retention of knowledge in this study may be explained by the active use of AI for repeated questioning, clarification, and retrieval practice, rather than passive access to information alone.

The quantitative and qualitative findings converge in a way that strengthens confidence in these results. The Method × Time interaction (F(1.46, 137.43) = 62.23, p < 0.001) showed that the AI-assisted advantage was not constant but grew from immediate post-test (d = 0.68) to a peak at one-week retention (d = 1.39) before narrowing at one-month (d = 1.01). This trajectory maps closely onto the qualitative theme of active engagement: students with higher retention scores described iterative, self-paced questioning of the AI system, exemplified by the statement, "Repeated prompts at my own pace made me remember because it became active learning." Such a pattern of active retrieval practice would be expected to produce a delayed, retrieval-dependent advantage of this shape, rather than an immediate and uniform one. Conversely, the cognitive offloading theme, in which some students passively copied AI-generated answers, offers a plausible qualitative explanation for why the group-level advantage narrowed rather than continued to widen at one month: a subset of students who engaged superficially with the AI content may have diluted the aggregate retention advantage at the final timepoint, even as students who engaged actively continued to benefit.

AI-assisted learning in relation to Bloom's 2 Sigma framework

Using a Bloom-inspired heuristic benchmark, 43.4% of students in AI-assisted learning exceeded the predefined threshold compared with 3.0% of traditional learning. Ferreira has explained the GenAI model in the context of Bloom's 2 Sigma problem. He proposed that AI agents can simulate one-to-one tutoring for students in many aspects by providing adaptive explanations, tailored feedback, scaffolded learning, and iterative learner interaction [13]. Similarly, Kasneci and colleagues emphasised that AI models have the potential to transform education by supporting personalized, learner-centered instruction [1]. The higher proportion of students achieving the Bloom-inspired benchmark following AI-assisted learning suggests that AI-assisted learning may reproduce some educational characteristics of individualized tutoring. However, this should not be interpreted directly as an illustration of the original Bloom's 2 Sigma effect. Students also expressed their experience with AI as a personal tutor, which came under one of the themes.

Recent studies suggest that AI tutoring could reach Bloom's 2 Sigma effect since they give adaptive explanations based on learners' pace, mastery-based progression, immediate feedback, and better learner engagement. Instead of giving just answers, the AI tutors give concepts step by step till mastery is achieved. This could be the reason for deeper understanding and long-term memory. These concepts closely resemble the findings and perceptions proposed by this study [14,15].

AI-assisted learning and long-term memory retention

In the present study, forgetting was greater with traditional learning than with AI-assisted learning. The Ebbinghaus forgetting curve describes the rapid loss of newly acquired information unless it is reinforced by retrieval mechanisms [7]. Radvansky et al. further explained that forgetting is a dynamic process influenced by opportunities for the retrieval process and not just by the passage of time [16]. So, retrieval is a more important process for memory consolidation [17]. This is supported by the qualitative findings of the role of retrieval practice in AI-assisted learning. With regard to the high retention of knowledge, students described that they repeatedly questioned the AI, sought clarification, compared clinical explanations, and revisited difficult concepts until they understood them completely [18].

The immediate, repeated, and non-threatening feedback provided by AI could improve retrieval by correcting misunderstandings as and when they occur, promoting consolidation of memory. Rather than passive review, the timely feedback encourages retrieval and refinement to improve memory retention [4,17,18].

An alternative explanation for the better retention noted with AI-assisted learning is the novelty effect: the responsive, conversational nature of generative AI may have increased student interest and engagement simply because it was new and unfamiliar, rather than because of any inherent pedagogical advantage. The two-way interaction, immediate responses, and scope for repeated questioning may have increased active involvement compared with the traditional study method regardless of instructional content. This explanation is difficult to fully separate from genuine pedagogical benefit within a single-exposure, non-crossover design, since students encountered the AI-assisted format for the first time during the study. The narrowing of the effect size between one-week (d = 1.39) and one-month retention (d = 1.01) is compatible with, though not conclusive evidence for, partial novelty decay over the follow-up period. A crossover design with repeated exposure to both modalities, or a longer follow-up interval, would be needed to more clearly separate a durable pedagogical effect from an initial novelty-driven engagement effect. The qualitative finding that engagement quality varied markedly between students, active questioners versus passive copiers, also argues against a pure novelty account, since a novelty effect alone would not be expected to produce such divergent patterns of use within the same newly introduced tool.

Conceptual understanding and multimedia learning

With simple prompts, students can engage with an AI model to receive repeated explanations, flowcharts, concept maps, comparisons, and clinical correlations rather than memorizing isolated pharmacological facts, which favours the meaningful conceptual understandings of the subject. This is coherent with Ausubel's theory of meaningful learning, which highlights that long-term retention is enriched when new knowledge is meaningfully integrated with existing cognitive structures [19]. These findings are further supported by Chi's Active-Constructive-Interactive framework [20].

Participants also reported that tables, pictures, flowcharts, and visual summaries generated by AI helped them remember the concepts more effectively. This is supported by Paivio's Dual Coding theory: when information is presented through both verbal and visual formats, the ability of retention is higher than the single mode of presentation [21]. Similarly, Mayer's Cognitive Theory of Multimedia Learning also supports this evidence, suggesting that blending text with suitable visual representations improves understanding and long-term retention [22].

The above findings can also be explained by the cognitive load theory. Pharmacology is an intrinsically complex subject integrating drug classifications, mechanism of action, pharmacological actions, therapeutic uses, and adverse reactions. AI-assisted learning could have reduced intrinsic load by reducing complex concepts to simpler explanations, flowcharts, and clinical reasoning. Also, individualized explanations and retrieval may have enhanced germane load by promoting mental schema formation. All these mechanisms may have contributed to long-term retention noted following AI-assisted learning [23,24].

Apart from multimedia presentation, AI tutoring also enhances scaffolded learning by modifying questions based on students' questions and understanding. This scaffolding reduces cognitive load and facilitates meaningful learning rather than rote memorization. This may explain why students observed AI assistance as a personal tutor rather than just an information provider [4,18,22].

Active engagement versus cognitive offloading

Although there was significant retention of knowledge, the qualitative findings also emphasized the risk of cognitive offloading. In the present study, students with lower retention scores reported that they passively read or copied AI-generated responses without actively processing the information. So, this may lead to cognitive offloading. This is supported by Sparrow et al., who described the "Google effect," suggesting that easy access to digital information may reduce the tendency to retain information internally [8]. Risko and Gilbert further proposed that individuals increasingly rely on external technologies as cognitive aids [9]. Firth et al. suggested that excessive dependence on digital technologies may influence attention, memory, and learning [25]. Therefore, generative AI has to be used actively; the answers have to be processed to avoid cognitive offloading.

The educational benefits of AI noted in this study should not be inferred as the advantage of unrestricted AI use. Studies suggest that AI-assisted learning is best when AI engagement is supported by better pedagogical scaffolding and supervision by faculty. Without guidance, learners may engage in superficial learning and cognitive offloading [1,9].

Comparison with emerging evidence on AI and cognition

Recently, the Massachusetts Institute of Technology (MIT)'s Your Brain on ChatGPT report proposed that independent thinking and memory may be reduced by using AI for essay writing [26]. However, the present study was different. Students used AI as a learning tool; they used it for clarifying doubts and understanding concepts. Then, there was an assessment of their learning, and here we got the contrary results. Therefore, if AI is used for just copying, the learning skills may be reduced. However, if it is used appropriately, the results on memory and learning outcomes are positive.

The MIT study analyzed essays, and the present study focused on VSAQs. The type of assessment may also influence the results of the studies. The contrasting results across studies suggest that the effectiveness of AI tools depends less on the technology itself than on the manner in which it is integrated into learning, consistent with the active-engagement versus cognitive-offloading distinction discussed above [8,9,20,26].

Strengths and limitations

This mixed-methods design combined qualitative and quantitative components, used a within-subject design to reduce inter-subject variability, and assessed retention at three timepoints; the Bloom-inspired heuristic benchmark offered a novel evaluative lens but should be read as an educational metric, not a replica of Bloom's original framework.

The main limitation is the non-randomized, fixed-order design: all students completed traditional learning before AI-assisted learning, with sessions separated by a minimum of one month, so test familiarity and general study-skill gains cannot be ruled out as alternative explanations; adherence to the no-AI instruction during the traditional session was also not independently verified. Randomized crossover designs would address this.

A second limitation is that different topics (aminoglycosides, quinolones) were used for the two modalities; baseline pre-test scores differed significantly (traditional: 6.34, AI: 4.73, P<0.001), indicating unequal prior familiarity, and the topic-specific VSAQ instruments were not formally equated for difficulty. A topic-reversed crossover design, or a single shared topic with psychometrically calibrated items, would separate instructional, familiarity, and instrument effects.

AI access between scheduled assessments was neither restricted nor logged, so retention scores may partly reflect undocumented AI-assisted revision rather than durable memory alone. Students used different AI platforms (ChatGPT, Gemini, NotebookLM) on personal devices without recording model versions, limiting reproducibility; findings should be read at the level of AI-assisted learning generally, not any specific platform. AI-generated content was not independently fact-checked, and several students described trusting AI output without verification (e.g., "I only read the answers"), raising the possibility that some retained knowledge was inaccurate--consistent with broader calls for AI literacy and verification safeguards [27,28].

Finally, the single-centre design, restriction to second-year MBBS students and VSAQ-based assessment, one-month follow-up ceiling, and lack of formal inter-rater reliability testing all limit generalizability; longer follow-up (3-6 months) and a dual-rating protocol are recommended for future work. Selection bias from non-random attrition is addressed in the Results. Completers did not differ from non-completers on baseline scores (p = 0.881, p = 0.975), arguing against ability-based attrition, though engagement-based selection cannot be excluded.

## Conclusions

In this quasi-experimental, mixed-methods study, AI-assisted learning was associated with higher immediate, one-week, and one-month retention scores compared with traditional learning under the specific conditions investigated. Because students were not randomized to modality and underwent both learning methods in a fixed sequence using different topics, this association cannot be interpreted as establishing that AI-assisted learning caused the improvement in retention; order effects, topic non-equivalence, and other threats to internal validity discussed above remain plausible alternative or contributing explanations. Qualitative findings suggested that students perceived AI as functioning like a personalized tutor and described features consistent with scaffolded, adaptive interaction; however, these are self-reported perceptions rather than a demonstration that AI-assisted learning replicates the mechanisms of individualized human tutoring, and the proportion of students exceeding the Bloom-inspired heuristic benchmark should be interpreted as a descriptive, exploratory observation rather than evidence of a Bloom's 2 Sigma-equivalent effect. Active engagement with AI, through repeated questioning and clarification, appeared more consistently linked to higher retention than passive use, which was associated with a risk of cognitive offloading.

Confirmation of these associations, and of any causal role for AI-assisted learning, will require randomized crossover trials using equivalent or counterbalanced topics, standardized AI platforms, assessment instruments validated and calibrated for difficulty across conditions, and complete participant-level analyses that account for attrition and selection effects.

## Appendices

Semi-structured interview guide for focus group discussions

Opening Questions

How was your overall experience using AI-assisted self-directed learning?

How did this experience differ from your conventional textbook-based learning?

Main Questions

What aspects of AI-assisted learning did you find most useful? Why?

Were there any features that helped you understand pharmacology concepts better? Please explain.

How did AI-assisted learning influence your ability to remember the topic during the one-week and one-month assessments?

What types of AI-generated outputs (e.g., explanations, flowcharts, tables, concept maps, mnemonics) did you find most helpful?

Were there any challenges or difficulties while using AI during self-directed learning?

Did you ever rely on AI-generated answers without verifying or fully understanding them? If yes, please describe your experience.

How did you verify the accuracy of AI-generated information?

Compared with conventional learning, what advantages and disadvantages did you perceive in AI-assisted learning?

How do you think AI can be used more effectively in undergraduate medical education?

Closing Questions

Would you recommend AI-assisted learning to future medical students? Why or why not?

Is there anything else about your experience that you would like to share?

Probing Questions (used whenever required)

Could you explain that further?

Can you give an example?

What made you feel that way?

Could you elaborate on that point?

Did anyone have a different experience?

Would anyone like to add something?
